# Evaluation of the Safety and Feasibility of Apheresis in Dogs: For Application in Metastatic Cancer Research

**DOI:** 10.3390/ani11102770

**Published:** 2021-09-23

**Authors:** Haru Yamamoto, Mohamed Elbadawy, Koudai Fujisaka, Yomogi Sato, Takahiro Ohmori, Yuta Shinohara, Yui Hatano, Daichi Kobayashi, Ayana Gomyo, Yuji Sudo, Daigo Azakami, Tsuyoshi Uchide, Ryuji Fukushima, Shohei Morita, Amira Abugomaa, Hideyuki Yamawaki, Masahiro Kaneda, Tatsuya Usui, Kazuaki Sasaki

**Affiliations:** 1 Laboratory of Veterinary Pharmacology, Cooperative Department of Veterinary Medicine, Faculty of Agriculture, Tokyo University of Agriculture and Technology, 3-5-8 Saiwai-cho, Fuchu, Tokyo 183-8509, Japan; s206628w@st.go.tuat.ac.jp (H.Y.); mohamed.elbadawy@fvtm.bu.edu.eg (M.E.); s162840t@st.go.tuat.ac.jp (K.F.); s202090x@st.go.tuat.ac.jp (Y.S.); y.shinohara@iskra.co.jp (Y.S.); s193249s@st.go.tuat.ac.jp (A.A.); skazuaki@cc.tuat.ac.jp (K.S.); 2 Department of Pharmacology, Faculty of Veterinary Medicine, Benha University, Moshtohor, Toukh 13736, Elqaliobiya, Egypt; 3 Animal Medical Center, Faculty of Agriculture, Tokyo University of Agriculture and Technology, 3-5-8 Saiwai-cho, Fuchu, Tokyo 183-8509, Japan; tkhromori5@gmail.com (T.O.); ryu-ji@cc.tuat.ac.jp (R.F.); fq7087@go.tuat.ac.jp (S.M.); 4 Pet Health & Food Division, Iskara Industry Co., Ltd., 1-14-2, Nihonbashi, Chuo-ku, Tokyo 103-0027, Japan; 5 Laboratory of Clinical Oncology, Cooperative Department of Veterinary Medicine, Faculty of Agriculture, Tokyo University of Agriculture and Technology, 3-5-8 Saiwai-cho, Fuchu, Tokyo 183-8509, Japan; s160384s@st.go.tuat.ac.jp (Y.H.); s167504x@st.go.tuat.ac.jp (D.K.); s179850u@st.go.tuat.ac.jp (A.G.); s170620w@st.go.tuat.ac.jp (Y.S.); 6 Laboratory of Veterinary Molecular Pathology and Therapeutics, Cooperative Department of Veterinary Medicine, Faculty of Agriculture, Tokyo University of Agriculture and Technology, Fuchu, Tokyo 183-8538, Japan; uchide@cc.tuat.ac.jp; 7 Faculty of Veterinary Medicine, Mansoura University, Mansoura 35516, Dakahliya, Egypt; 8 Laboratory of Veterinary Pharmacology, School of Veterinary Medicine, Kitasato University, 35-1, Higashi 23 Ban-cho, Towada, Aomori 034-8628, Japan; yamawaki@vmas.kitasato-u.ac.jp; 9 Laboratory of Veterinary Anatomy, Cooperative Department of Veterinary Medicine, Faculty of Agriculture, Tokyo University of Agriculture and Technology, 3-5-8 Saiwai-cho, Fuchu, Tokyo 183-8509, Japan; kanedam@cc.tuat.ac.jp

**Keywords:** apheresis, circulating tumor cells, metastasis, MCF7 cells, monocyte, dogs

## Abstract

**Simple Summary:**

In cases of cancer metastasis, some tumor cells circulate in blood (CTCs) and function as a seed for metastasis. These cells are important for detailed study of the molecular basis of metastasis. However, their few numbers and difficult isolation hinder its analysis. Apheresis, a process used to isolate a specific component of blood, can efficiently separate peripheral blood monocyte (PBMC), whose density is closely like that of CTCs. In the present study we used the dogs as a precious model of human’s cancers to check the safety and feasibility of apheresis and to separate PBMC and infused MCF7 cells using Spectra Optia apheresis machine (Terumo). The process was performed safely to capture PBMC and MCF7. The captured MCF7 cells were regrown up in vitro and characterized. In conclusion, Spectra Optia apheresis machine (Terumo) can be used safely to isolate CTCs from dogs blood with precautions to keep hemodynamic stability.

**Abstract:**

In patients with solid tumors, circulating tumor cells (CTCs) spread in their blood and function as a seed for metastases. However, the study of CTCs has been limited by their rarity, low frequency, and heterogeneity. The efficient collection of CTCs will contribute to further research of metastatic cancers. Apheresis is a process in which the whole blood of an individual is passed through a machine that isolates a particular constituent and returns the remainder to the circulation. In the present study, we investigated the safety and feasibility of apheresis to separate peripheral blood monocytes (PBMCs), whose density is closely similar to that of CTCs, and to capture intravenously administered human breast cancer cells, MCF7s, from the dogs. No life-threatening events were observed in dogs during the apheresis process. The changes in the hemogram were transient and recovered gradually within a few days after apheresis. During apheresis, 50 mL of PBMCs could be collected from each dog. Notably, a thrombus was formed along the circuit wall during apheresis, which decreased the blood collection pressure. MCF7 cells were successfully captured by the apheresis machine. The captured cells were regrown in vitro and characterized compared with the original cells. In conclusion, apheresis could be safely performed in dogs to isolate CTCs with precautions to maintain hemodynamic stability.

## 1. Introduction

In cases of cancer metastasis, tumor cells often circulate in the blood or lymph stream. They are called circulating tumor cells (CTCs) and function as a seed for metastasis in other organs. Recently, CTCs have attracted more attention to study the molecular basis of different metastatic cancers due to their potential role in early diagnosis [1,2], prognosis [3,4], monitoring of the therapeutic response to anti-cancer drugs, and drug development [4,5,6] in cancer-diseased patients. However, the low number and difficult isolation of CTCs hinder their laboratory and clinical investigation. It is known that the density of CTCs is closely similar to that of peripheral blood monocytes (PBMCs) [7]. Apheresis, a medical process that is used to separate a specific constituent of patients’ blood using certain machines, can efficiently separate PBMCs. Therefore, it was recently employed to capture CTCs in non-metastatic [8] and metastatic cancer-diseased patients. Furthermore, apheresis is used therapeutically to treat various health disorders, including hematological, oncological, and autoimmune diseases [9,10,11,12,13,14]. Compared to other methods that use a small volume of blood to isolate fewer CTCs [15,16,17], apheresis, which uses the whole blood, could capture several hundreds of CTCs to be used for molecular analysis of their parental tumors [8,18,19].

Spontaneous cancers in dogs closely recapitulate human ones regarding histological structure, molecular imprints, clinical aspects, aggressiveness, metastasis, and response and resistance to therapy [20,21,22,23]. Therefore, dogs are valuable models for the study of the molecular mechanisms of cancer, including invasion and metastasis. Dog models have been used to isolate and study CTCs using different isolation techniques. Kim et al. developed an in vivo in-dwelling apheresis system for continual collection of CTCs directly from a peripheral vein [24]. However, it took over 2 h to screen 1–2% of the whole blood, and the technology used in the study is still not available commercially. In a different manner, Wright et al. have developed flow cytometric detection of intracellular collagen I and osteocalcin in blood samples from tumor-bearing dogs for the detection of circulating osteosarcoma cells [25].

Diagnostic leukapheresis of a large volume of blood with continuous centrifugation and the return of all blood components, except the mononuclear cell fraction including CTCs, was recently developed to enrich the number of isolated CTCs [8,26]. Few instances of apheresis have been attempted in dogs to isolate PBMC cells. The safety of apheresis machines, including the COBE Spectra Apheresis System (Gambro BCT, Lakewood, CO) [27] and CaridianBCT apheresis machines [28], was assessed in dogs to isolate PBMCs, and, later, apheresis machines such as COBE Spectra and Spectra Optia (Terumo BCT, Lakewood, CO, USA) were used successfully in dogs to isolate PBMCs in preparation for hematopoietic stem cell transplantation [29]. However, little is known about using apheresis machines to separate CTCs in dogs. The objectives of the present study were to investigate the safety and feasibility of apheresis in dogs to separate PBMCs whose density is closely similar to that of CTCs using Spectra Optia (Terumo) set to continuous mononuclear cell collection (CMNC) mode. We verified the best conditions for the apheresis system to avoid complications and hemodynamic instability. Furthermore, the entire system was tested to mimic the capture of CTCs from dogs using an MCF7 infusion. The cells were intravenously administered, captured, regrown in vitro, and analyzed in comparison to the non-injected original cells. 

## 2. Materials and Methods

### 2.1. The Spectra Optia Apheresis System

The Spectra Optia system (Terumo BCT, Lakewood, CO, USA) was recently introduced to replace the COBE Spectra apheresis system, which had been the most frequently used apheresis device for several decades. It is a safe and efficient system for hematopoietic progenitor cell apheresis and is provided with an advanced automated interface management system that allows operators to spend more time focusing on patient care [30]. Furthermore, it can automatically detect and maintain a buffy coat interface for continuous PBMC collection and transfer it into a collection bag with a lower product hematocrit (HCT%) [31].

### 2.2. MCF7 Cells and Antibodies

MCF7 cells, a human breast adenocarcinoma cell line of metastatic origin with multi-receptor expression of estrogen, progesterone, androgen, and glucocorticoid receptors [32], were purchased from the American Type Culture Collection (ATCC, University Boulevard, Manassas VA, USA). Cells were cultured in Eagle’s minimum essential medium with non-essential amino acids, 1 mM sodium pyruvate, and 0.01 mg/mL insulin with 10% fetal bovine serum and maintained at 37 °C with 5% CO_2_. After the confluent condition, cells were used to mimic the in vivo capture of CTCs using an apheresis machine. After cell isolation, cells were cultured and characterized using the primary antibody, anti-pan-cytokeratin antibody (AE1/AE3, 1:200, Novus Biologicals, Centennial, CO, USA). The fluorescent secondary antibodies used were Alexa Fluor 488™ goat anti-mouse IgGs (Thermo Fisher Scientific Inc., Waltham, MA, USA).

### 2.3. Animals

Five female beagle dogs of 9–10 kg (mean 10 kg) and 6 years of age were purchased from the Research Institute for Animal Science in Biochemistry and Toxicology (Kanagawa, Japan), at which they were regularly treated with anthelmintic drugs and immunized against common viral diseases of pets. The protocol of the apheresis process was approved by the Ethics Committee of Tokyo University of Agriculture and Technology Animal Hospital (Approval number; R02-70). The dogs underwent physical examination, blood screening, biochemical examination, and chest X-ray to ensure their soundness. Two weeks before the experiment, all dogs were kept in appropriate cages at 22 °C and 65% humidity with a 12 h light/dark cycle. Three dogs underwent PBMC apheresis at the small animal medical center of the university to evaluate the safety and feasibility of apheresis in dogs to separate PBMCs whose density is closely similar to that of CTCs. Under the same apheresis condition, two dogs were used for MCF7 infusion and isolation experimentation.

### 2.4. Preparation of Dogs for Apheresis

Priming the system of the apheresis machine with normal saline followed by autologous blood is necessary to ensure hemodynamic stability during PBMC collection. Therefore, two weeks before the experiment, about 200 mL autologous blood (20 mL/kg) was collected from each dog in a sterile blood-collection bag with acid–citrate–dextrose formula A (ACD-A, Terumo BCT) and kept at 4 °C until the day of apheresis. Before this sampling, the dogs were sedated with atipamezole 10~20 µg/kg (Nippon Zenyaku Kogyo Co., Fukushima, Japan) and awakened with medetomidine 10~20 µg/kg (Nippon Zenyaku Kogyo Co., Fukushima, Japan). The food was withheld from all dogs 12 h before the apheresis process, while water was provided ad libitum. On the day of apheresis, to customize the length of the PBMC apheresis process, individual data (HCT%, body weight, height ≥ 80 cm, and gender) were collected to establish each dog’s total blood volume (TBV) and its optimal pump rates considering the flow rate of the anticoagulant (AC) [33].

### 2.5. Procedure of Apheresis

Under general anesthesia, the apheresis was performed using the Spectra Optia system set to CMNC mode. A 22G catheter was placed in the flexor cutaneous vein and physiological saline (Otsuka Pharmaceutical Co., Tokyo, Japan) was administered through it to dogs at 3~5 mL/kg/h. This saline was used to administer the intravenous (i.v.) anesthetics and to maintain body conditions at the optimal state. For anesthesia, 0.022 mg/kg atropine (Mitsubishi Tanabe Pharma Co., Osaka, Japan) was injected subcutaneously (s.c.) as a pre-anesthetic medication followed by i.v. injection of 0.2 mg/kg midazolam (Astellas Pharma, Tokyo, Japan) and 6~8 mg/kg propofol (Zoetis, Tokyo, Japan). In dog 3, 0.2 mg/kg butorphanol (Meiji Seika Pharma, Tokyo, Japan) was i.v. administered instead of midazolam. After that, tracheal intubation was performed, and anesthesia was maintained using isoflurane (DS Pharma Animal Health, Tokyo, Japan) in oxygen to maintain the state of spontaneous respiration.

After priming the machine with saline, the collection bag containing 200 mL of autologous blood was connected and the apheresis procedure was started. After the collection bag became nearly empty, the procedure was temporarily paused, and the dogs were then connected to the apheresis machine. Before cannulation, the puncture site was shaved, cleaned, and sterilized with an antiseptic solution. The blood removal and returning catheters were connected to the apheresis machine and the process was resumed. For blood removal from the animal to the apheresis machine, a 16-gauge, 30 cm single-lumen central venous catheter (Terumo, CVC) was placed in the jugular vein as previously described [34]. For returning blood from the apheresis circuit to the animal, a 20-gauge catheter was placed in the saphenous vein.

During apheresis, the anticoagulant (ACD-A) was administered at a rate of 0.8 mL/minute/L of TBV to prevent blood clotting. The best position of the PBMC collection orifice was set by changing the setting of collection preference (within the range of 50–70) so that the color of the harvested product appeared whitely opaque and not showing the color of excessive RBC contamination. The inlet flow rate was set at 14 to 16 mL/min. Electric heating pads were used to maintain the body temperature of dogs in the physiological range. To prevent hypotension and hypocalcemia secondary to ACD-A use, continuous infusion of 8.5% calcium gluconate (Nichi-Iko Pharmaceutical Co., Japan) at 0.1~0.5 mL/kg/h and dopamine hydrochloride (Kyowa Hakko Kirin Co., Tokyo, Japan) at 3~5 µg/kg/min was performed [35]. Dopamine was diluted by a 5% glucose solution. The anesthesia depth, heart rate, temperature, blood pressure, respiratory rate, and oxygen saturation were monitored every 5 min. In addition, CBC, and serum levels of calcium, sodium, and potassium were monitored hourly during the treatment. Table 1 summarizes the parameters of the apheresis.

The remaining blood and fluid (~450 mL) in the system of the machine (rinseback) were collected into a transfusion collection bag (Terumo) and centrifuged at 2000× *g* for 15 min to remove the plasma. The obtained ~150 mL of packed red blood cells were transfused back into the dog over ~4 h. Thereafter, catheters were aseptically removed, and adhesive bandages were placed at the puncture sites for 15 min to ensure hemostasis; a sterile dressing and an Elizabethan collar were maintained for 24 h.

### 2.6. Management after Apheresis

Further blood samples were taken immediately after and on the 3rd and 5th day following apheresis for CBC analysis and monitoring of serum calcium, sodium, and potassium.

### 2.7. Comparison of PBMC Concentration Rate

The mononuclear cells collected by blood sampling from the flexor cutaneous vein and by apheresis were compared using the concentration rate. The formula used to calculate the concentration rate is shown as follows:

PBMC concentration (%)=( Lymphocyte (KµL)+Monocyte (KµL))White Blood Cell (KµL)×100

### 2.8. Cell Culture and Characterization after Apheresis

The feasibility of our approach was validated to mimic the natural incidence of CTCs in blood and to interpret the concept of in vivo CTC capture using an apheresis system. Before cell infusion, apheresis was run for 30 min to establish the optimum conditions. The process was continued to isolate PBMCs. Subsequently, a total of 1 × 10^7^ MCF7 cells were suspended in one mL phosphate-buffered saline (PBS) and infused into the circulation of the dogs using the saphenous vein. The collection of PBMCs was started after one minute of the infusion and continued for 90 min to capture the circulating MCF7 cells, as indicated [24]. Captured MCF7 cells were immediately washed in PBS and cultured in cell culture media [23]. The cultured cells were characterized using AE1/AE3 antibody (1:200) and compared to the original MCF7 cells before injection.

### 2.9. Immunofluorescence Staining

Immunofluorescence staining of cultured cells was performed as described previously [36]. After fixation in 4% paraformaldehyde (PFA) for 15 min, cells were blocked with 1.5% normal goat serum (NGS)/PBS at room temperature for 1 h. Subsequently, they were incubated with primary antibodies (AE1/AE3, 1:200) and kept at 4 °C overnight. Thereafter, the cells were washed 3 times with PBS and incubated with the secondary antibody for 1 h. The images were captured by the fluorescence microscope (BX-52; Olympus, Tokyo, Japan).

### 2.10. Data Analysis

Statistical analysis was performed using SigmaPlot (V 14.5, Systat Software Inc., San Jose, CA, USA). The PBMC concentration rate in the collection obtained from pre-apheresis and in the collection bag after apheresis in dogs was compared. To detect differences in concentration rate, one-way analysis of variance followed by Bonferroni’s test was performed. *p*-values < 0.05 were considered statistically significant.

## 3. Results

### 3.1. Effects of Apheresis on Blood Parameters

**Figure 1:**
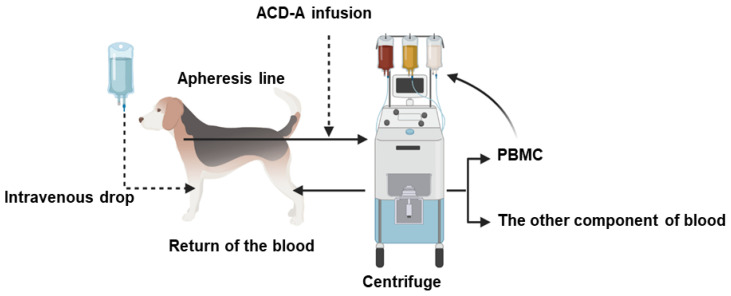
The procedure of apheresis in dogs. Blood was removed from the jugular vein and returned to the lateral saphenous vein. The peripheral blood mononuclear cells (PBMCs) obtained by apheresis were collected in a collection bag. During the procedure, ACD-A, saline, dopamine, and calcium gluconate were given intravenously.

**Figure 2:**
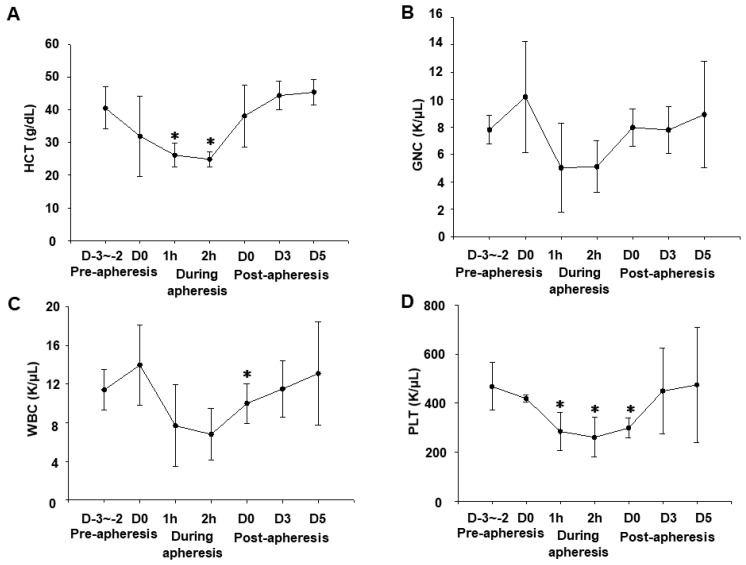
The transition of the values of hematocrit (HCT), granulocytes (GNCs), white blood cells (WBCs), and platelets (PLTs) in dogs. Blood samples were taken from the cephalic vein in pre-apheresis (day (D)-3~2), during apheresis (1, 2 h), and post-apheresis time (D0, 3, 5). Values of HCT (**A**), GNC (**B**), WBC (**C**), and PLT (**D**) were analyzed. Data were shown as mean ± S.E.M (*n* = 3). ∗ *p* < 0.05 vs. D-3~-2 pre-apheresis.

Since the apheresis process is to remove blood from the patients, centrifuge it in the device, and return blood except for the required component (Figure 1), it is essential to monitor the changes of blood cell components such as HCT% and platelets (PLTs). We, therefore, compared the changes in blood cell components, HCT (Figure 2A), granulocytes (GNCs) (Figure 2B), white blood cells (WBCs) (Figure 2C), and PLTs (Figure 2D). Although there were no major abnormalities in the pre-apheresis blood test, there was a decrease in the HCT value in some dogs that reached 20% from the pre-apheresis level. However, 5 days after the apheresis, these parameters returned to their normal level.

### 3.2. Effects of Apheresis on Serum Calcium, Sodium, and Potassium Levels

**Figure 3:**
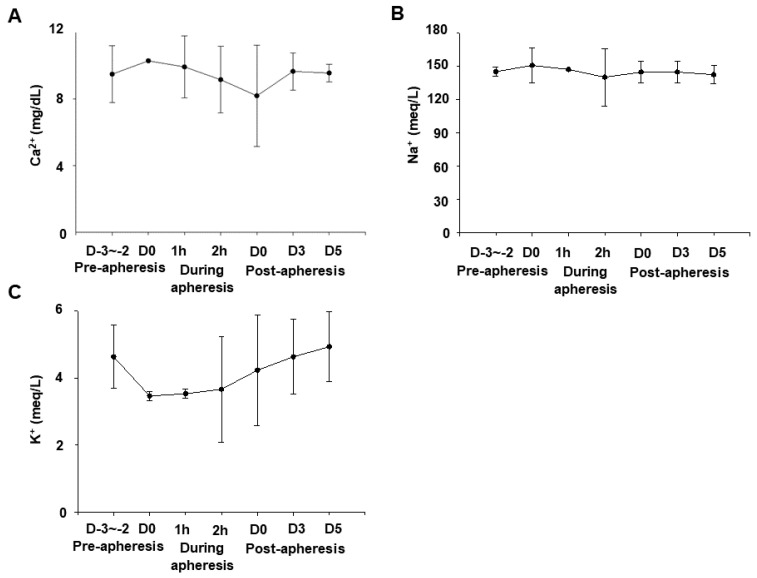
The transition of the blood concentrations of calcium (Ca^2+^), sodium (Na^+^), and potassium (K^+^) in dogs. Blood samples were taken from the cephalic vein pre-apheresis (D-3~2), during apheresis (1, 2 h), and post-apheresis (D0, 3, 5). Values of Ca^2+^ (**A**), Na^+^ (**B**), and K^+^ (**C**) were analyzed. Data were shown as mean ± S.E.M (*n* = 3).

Since the ACD-A solution, which was used as AC during blood flow to the apheresis machine, returns to the body with the blood, it can possibly chelate the blood calcium and cause hypocalcemia. Additionally, we used saline to maintain the blood flow and blood pressure during general anesthesia. Therefore, we monitored serum concentrations of calcium (Ca^2+^), sodium (Na^+^), and potassium (K^+^) pre-, during, and post-apheresis. There were no significant changes in their normal levels as shown in Figure 3A–C, respectively. These data indicate that the apheresis process did not affect the level of these important electrolytes in the blood of these dogs.

### 3.3. Efficacy of Mononuclear Cell Collection by Apheresis

**Figure 4:**
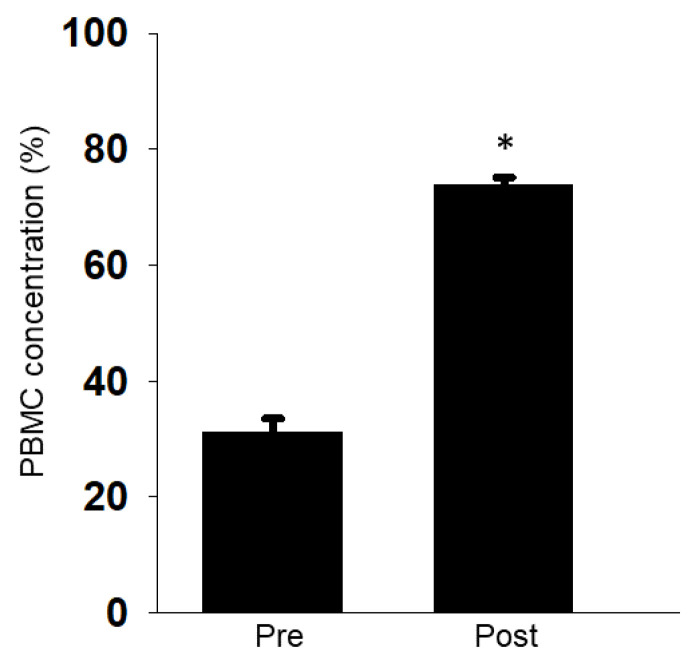
Collection efficacy of PBMCs by apheresis. PBMC concentration pre-apheresis (Pre) and post-apheresis (Post) was calculated. Results were expressed as mean ± S.E.M (*n* = 3). ∗ *p* < 0.05 vs. Pre.

Next, we checked whether the apheresis efficiently collects PBMCs in dogs. The collection rate of PBMCs was compared based on the sum of lymphocytes (Lym) and monocytes (Mono) in the WBCs obtained by blood sampling or the apheresis. Before the apheresis, PBMCs accounted for 20–40% of WBCs in the peripheral blood of the three dogs tested. After performing apheresis, the PBMCs accounted for 70 to 75% of the WBCs collected by the apheresis process (Figure 4).

### 3.4. Occurrence of a Thrombus during Apheresis

All dogs tolerated the apheresis process using the Spectra Optia device with minimal adverse events. During apheresis, we found that the blood collection pressure from the jugular vein gradually decreased and the device stopped. At that time, we confirmed that a thrombus formation along the circuit wall was observed between the external apheresis circuit and the blood removal route. To remove the thrombus, we flushed with saline containing heparin. After reconnecting the circuit, the device returned to regular operation. It was observed that the blood collection pressure gradually increased after the removal of the thrombus (data not shown).

### 3.5. Collection of Intravenously Infused Cancer Cells by Apheresis

**Figure 5:**
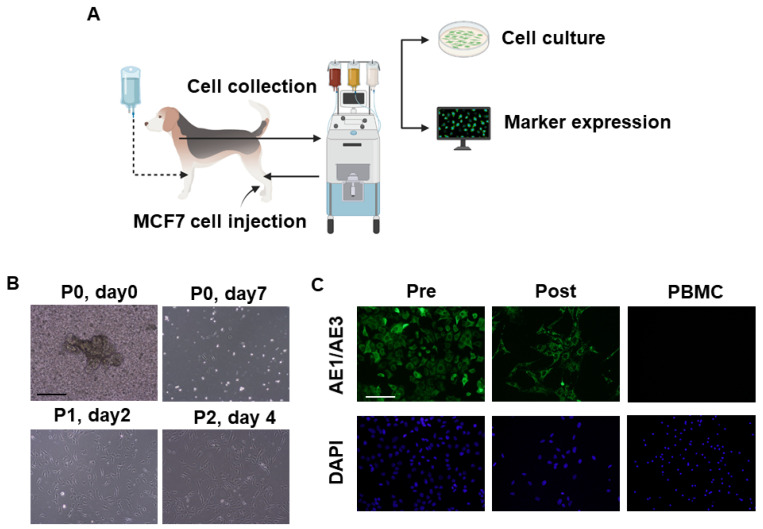
Cancer cell collection by apheresis in dogs. Schematic illustration of MCF7 injection, collection, culture, and characterization (**A**). Representative phase-contrast images of MCF7 cells after collection by apheresis (n = 2). Scale bar: 100 μm (**B**). Representative fluorescence images of epithelial cell marker, AE1/AE3 expression in MCF7 cells pre- and post-apheresis as well as in dog PBMC cells. Scale bar: 100 μm (**C**).

After the intravenous infusion of MCF7 cells into each dog’s circulation, the apheresis machine successfully isolated the infused cells (Figure 5A,B). The captured cells were then cultured. However, few cells were attached (Figure 5B). The attached cells continued to grow (Figure 5B, upper panel). After confluence, cells were successfully passaged and propagated (Figure 5B, lower panel). Finally, we observed expression of the epithelial cell marker, AE1/AE3 in the cultured cells pre- and post-apheresis (Figure 5C). As a negative control, we prepared dog PBMC samples that were stained with AE1/AE3 antibody, and no staining was observed (Figure 5C). The results suggest the ability of this method to successfully capture the CTCs from the circulation and enable the subsequent morphological and molecular characterization of these cells, which is considered a valuable tool for studying the pathogenesis of cancer metastasis.

## 4. Discussion

In the present study, we showed that apheresis could be safely performed in dogs by the regular monitoring of blood pressure, respiration, calcium, HCT% (Figure 2 and Figure 3), and the appropriate maintenance of anesthesia. Moreover, the apheresis process yielded a higher concentration of PBMCs than regular whole blood sampling (Figure 4). It was suggested that the removal of intra-device clotting and intravascular thrombosis is essential for more efficient apheresis. Furthermore, the entire system was used successfully for the isolation of intravenously infused MCF7 cells as CTCs (Figure 5).

The apheresis causes acute blood loss due to the temporary flow of blood out of the body, which can lead to anemia. A decrease in HCT was observed during the process in all dogs, but immediately after the end, two out of the three dogs returned to their normal values, and the blood tests in the next day to apheresis showed normal ranges for the three dogs (Figure 2). To prevent hypotension, intravenous saline was administered in addition to dopamine. There have been some reports of hematological changes including anemia during apheresis that rapidly returns to normal levels [28,35]. Although the device system was primed with 200 mL autologous blood to maintain the HCT during apheresis, the original blood components may be diluted due to the influence of the coagulant solution in the blood bag, resulting in the obtained transient mild anemia. This phenomenon may become a problem when considering clinical application in cancer-diseased or critically ill animals. Therefore, further improvements such as the reduction in the infusion volume are required.

During apheresis, ACD-A solution was used to prevent thrombosis. Nevertheless, using a larger volume of ACD-A solution can chelate calcium when it enters the body with the returned blood from the machine, leading to hypocalcemia. Therefore, calcium preparations were infused at the same time with the physiological saline to prevent hypocalcemia. In human medicine, using a large-capacity leukocyte apheresis system in underweight children usually leads to hypotension, hypocalcemia, and low circulating blood volume. In the present experimental conditions, immediately after the apheresis, especially for dog 2 and dog 3, the serum calcium values were temporarily decreased, while the subsequent screening test showed gradually increasing values toward normal levels (Figure 3A). Furthermore, there were no symptoms of arrhythmia, prolonged QT interval, or bradycardia, which are commonly associated with hypocalcemia. Hypocalcemia may occur due to the use of ACD-A as an anticoagulant (AC) in large volumes, which can lead to electrolyte imbalances [37], but it can be avoided by infusion of calcium during the apheresis. The citrate-caused hypocalcemia mainly depends on the inlet flow, AC flow, and AC ratio [38]. Therefore, the parameters related to ACD-A need to be adjusted according to the serum calcium concentration. Heparin may also be used as an AC in apheresis and hemodialysis [38]. The usage of a heparin/citric acid combination in apheresis in children is limited, suggesting that it is necessary to select an AC suitable for each disease in dogs.

At the connection between the CVC and the apheresis machine, which is the blood removal route and the extracorporeal circulation circuit, the formation of a thrombus along the circuit was observed. Blood coagulation and clots during apheresis have been observed in sheep [39] and dogs [24]. This major concern has been resolved by rigorous sterilization and continuous infusion of ACD-A solution. The CVC used in this experiment was coated with AC, but a thrombus formation was recognized. Thus, it is recommended to flush the circuit every 30 min and monitor the blood pressure not only before the start of apheresis but also during the procedure.

After setting up the optimum apheresis conditions, we validated the system to mimic the isolation of naturally occurring CTCs using the injection of MCF7 cells (Figure 5A). The device succeeded in identifying and capturing some of the circulating MCF7 cells. Although many cells were captured efficiently, few cells were attached, and it took several days for them to be confluent (Figure 5B). The isolated cells were characterized as the original cells using AE1/AE3 antibody, while PBMCs did not respond to the antibody (Figure 5C). The low attachment and slow growth rate in the first few days after seeding might be due to the targeting of MCF7 cells by the dogs’ immune systems since the dogs used in these experiments were healthy and immunocompetent and the human MCF7 breast cancer cells were xenogeneic to dog species. Thus, the cells will be eliminated naturally from the dogs’ blood and not be expected to colonize. In a recent study, after the infusion of MCF7 cells into immunocompetent dogs, the cells were naturally eliminated from the dogs’ blood with an observed half-life of 2 h, and the recovery rate of MCF7 cells from the dogs’ blood decreased with time after the injection [24]. It is noteworthy that the dissemination rate of naturally occurring CTCs from cancer tumors differs depending on many factors including the patient’s condition, tumor type, and cancer stage. Therefore, the number of CTCs might differ from the homogenous distribution of CTCs used in the present study.

In conclusion, the described protocol showed that apheresis can be performed with safety and stability in healthy dogs and enable the collection of PBMCs with CTCs. However, a thrombus formation in the circuit was recognized as an adverse event. Further studies on the conditions for CTC collection will be suggested to contribute to the understanding of the pathophysiology and treatment of metastatic cancer-diseased animals.

## Figures and Tables

**Table 1:** Summary of PBMC apheresis parameters in dogs using Spectra Optia.

	BW (kg)	Sex	TBV (mL)	Total Length (min)	AC Flow (mL/min)	AC Volume (mL)	Inlet Flow (mL/min)	Inlet Volume (mL)	Plasma Flow (mL/min)	Collection Flow (mL/min)
Dog 1	11	female	990	154.0	0.9	138.6	10.8	1660.1	5.7	0.7
Dog 2	9	female	810	133.0	0.9	119.7	11.0	1463.0	5.4	0.5
Dog 3	10	female	900	153.0	0.9	131.1	10.3	1569.3	5.4	0.5

BW: body weight; TBV: total blood volume; AC: anticoagulant.

## Data Availability

All relevant data are included in the manuscript.

## References

[1] Alix-Panabieres C., Pantel K. (2013). Circulating tumor cells: Liquid biopsy of cancer. Clin. Chem..

[2] Peeters D.J., Brouwer A., Van den Eynden G.G., Rutten A., Onstenk W., Sieuwerts A.M., Van Laere S.J., Huget P., Pauwels P., Peeters M. (2015). Circulating tumour cells and lung microvascular tumour cell retention in patients with metastatic breast and cervical cancer. Cancer Lett..

[3] Cristofanilli M., Budd G.T., Ellis M.J., Stopeck A., Matera J., Miller M.C., Reuben J.M., Doyle G.V., Allard W.J., Terstappen L.W. (2004). Circulating tumor cells, disease progression, and survival in metastatic breast cancer. N. Engl. J. Med..

[4] Cohen S.J., Punt C.J., Iannotti N., Saidman B.H., Sabbath K.D., Gabrail N.Y., Picus J., Morse M., Mitchell E., Miller M.C. (2008). Relationship of circulating tumor cells to tumor response, progression-free survival, and overall survival in patients with metastatic colorectal cancer. J. Clin. Oncol. Off. J. Am. Soc. Clin. Oncol..

[5] Urtishak S., Alpaugh R.K., Weiner L.M., Swaby R.F. (2008). Clinical utility of circulating tumor cells: A role for monitoring response to therapy and drug development. Biomark. Med..

[6] Smerage J.B., Barlow W.E., Hortobagyi G.N., Winer E.P., Leyland-Jones B., Srkalovic G., Tejwani S., Schott A.F., O’Rourke M.A., Lew D.L. (2014). Circulating tumor cells and response to chemotherapy in metastatic breast cancer: SWOG S0500. J. Clin. Oncol. Off. J. Am. Soc. Clin. Oncol..

[7] Li X.C., Li Y., Shao W.Q., Li Z., Zhao R., Ye Z.L. (2020). Strategies for enrichment of circulating tumor cells. Transl. Cancer Res..

[8] Fischer J.C., Niederacher D., Topp S.A., Honisch E., Schumacher S., Schmitz N., Zacarias Fohrding L., Vay C., Hoffmann I., Kasprowicz N.S. (2013). Diagnostic leukapheresis enables reliable detection of circulating tumor cells of nonmetastatic cancer patients. Proc. Natl. Acad. Sci. USA.

[9] Padmanabhan A., Connelly-Smith L., Aqui N., Balogun R.A., Klingel R., Meyer E., Pham H.P., Schneiderman J., Witt V., Wu Y. (2019). Guidelines on the Use of Therapeutic Apheresis in Clinical Practice—Evidence-Based Approach from the Writing Committee of the American Society for Apheresis: The Eighth Special Issue. J. Clin. Apher..

[10] Connelly-Smith L., Dunbar N.M. (2019). The 2019 guidelines from the American Society for Apheresis: What’s new?. Curr. Opin. Hematol..

[11] Bouhairie V.E., Goldberg A.C. (2015). Familial hypercholesterolemia. Cardiol. Clin..

[12] Lambros M.B., Seed G., Sumanasuriya S., Gil V., Crespo M., Fontes M., Chandler R., Mehra N., Fowler G., Ebbs B. (2018). Single-Cell Analyses of Prostate Cancer Liquid Biopsies Acquired by Apheresis. Clin. Cancer Res. Off. J. Am. Assoc. Cancer Res..

[13] Klingele M., Allmendinger C., Thieme S., Baerens L., Fliser D., Jan B. (2020). Therapeutic apheresis within immune-mediated neurological disorders: Dosing and its effectiveness. Sci. Rep..

[14] Aguirre-Valencia D., Naranjo-Escobar J., Posso-Osorio I., Macia-Mejia M.C., Nieto-Aristizabal I., Barrera T., Obando M.A., Tobon G.J. (2019). Therapeutic Plasma Exchange as Management of Complicated Systemic Lupus Erythematosus and Other Autoimmune Diseases. Autoimmune Dis..

[15] Nagrath S., Sequist L.V., Maheswaran S., Bell D.W., Irimia D., Ulkus L., Smith M.R., Kwak E.L., Digumarthy S., Muzikansky A. (2007). Isolation of rare circulating tumour cells in cancer patients by microchip technology. Nature.

[16] Yoon H.J., Kozminsky M., Nagrath S. (2014). Emerging role of nanomaterials in circulating tumor cell isolation and analysis. ACS Nano.

[17] Paoletti C., Hayes D.F. (2016). Circulating Tumor Cells. Adv. Exp. Med. Biol..

[18] Tibbe A.G., Miller M.C., Terstappen L.W. (2007). Statistical considerations for enumeration of circulating tumor cells. Cytom. Part A J. Int. Soc. Anal. Cytol..

[19] Eifler R.L., Lind J., Falkenhagen D., Weber V., Fischer M.B., Zeillinger R. (2011). Enrichment of circulating tumor cells from a large blood volume using leukapheresis and elutriation: Proof of concept. Cytom. Part B Clin. Cytom..

[20] Gardner H.L., Fenger J.M., London C.A. (2016). Dogs as a Model for Cancer. Annu. Rev. Anim. Biosci..

[21] LeBlanc A.K., Mazcko C.N. (2020). Improving human cancer therapy through the evaluation of pet dogs. Nat. Rev. Cancer.

[22] Elbadawy M., Usui T., Mori T., Tsunedomi R., Hazama S., Nabeta R., Uchide T., Fukushima R., Yoshida T., Shibutani M. (2019). Establishment of a novel experimental model for muscle-invasive bladder cancer using a dog bladder cancer organoid culture. Cancer Sci..

[23] Abugomaa A., Elbadawy M., Yamanaka M., Goto Y., Hayashi K., Mori T., Uchide T., Azakami D., Fukushima R., Yoshida T. (2020). Establishment of 2.5D organoid culture model using 3D bladder cancer organoid culture. Sci. Rep..

[24] Kim T.H., Wang Y., Oliver C.R., Thamm D.H., Cooling L., Paoletti C., Smith K.J., Nagrath S., Hayes D.F. (2019). A temporary indwelling intravascular aphaeretic system for in vivo enrichment of circulating tumor cells. Nat. Commun..

[25] Wright T., Brisson B.A., Wood G.A., Oblak M., Mutsaers A.J., Sabine V., Skowronski K., Belanger C., Tiessen A., Bienzle D. (2019). Flow Cytometric Detection of Circulating Osteosarcoma Cells in Dogs. Cytometry. Part A J. Int. Soc. Anal. Cytol..

[26] Fehm T.N., Meier-Stiegen F., Driemel C., Jager B., Reinhardt F., Naskou J., Franken A., Neubauer H., Neves R.P.L., van Dalum G. (2018). Diagnostic leukapheresis for CTC analysis in breast cancer patients: CTC frequency, clinical experiences and recommendations for standardized reporting. Cytometry. Part A J. Int. Soc. Anal. Cytol..

[27] Lupu M., Gooley T., Zellmer E., Graves S.S., Storb R. (2008). Principles of peripheral blood mononuclear cell apheresis in a preclinical canine model of hematopoietic cell transplantation. J. Vet. Intern. Med..

[28] Posner L.P., Willcox J.L., Suter S.E. (2013). Apheresis in three dogs weighing <14 kg. Vet. Anaesth. Analg..

[29] Sekiguchi T., Vigani A., Ripoll A.Z., Taylor S., Culler C., Suter S.E. (2020). Clinical Application of Apheresis in Very Small Dogs Weighing <8 kg to Pediatric Patients. Ther. Apher. Dial..

[30] Even-Or E., Eden-Walker A., Di Mola M., McDougall E., Schechter T., Ali M., Svajger G., Gassas A., Licht C., Krueger J. (2017). Comparison of two apheresis systems for autologous stem cell collections in pediatric oncology patients. Transfusion.

[31] Pandey S., Cottler-Fox M. (2018). Optia(R) continuous mononuclear collection (CMNC) system is a safe and efficient system for hematopoietic progenitor cells-apheresis (HPC-a) collection and yields a lower product hematocrit (HCT%) than the COBE(R) spectra system: A retrospective study. J. Clin. Apher..

[32] Horwitz K.B., Costlow M.E., McGuire W.L. (1975). MCF-7; a human breast cancer cell line with estrogen, androgen, progesterone, and glucocorticoid receptors. Steroids.

[33] Nakasone H., Kanda Y., Ueda T., Matsumoto K., Shimizu N., Minami J., Sakai R., Hagihara M., Yokota A., Oshima K. (2009). Retrospective comparison of mobilization methods for autologous stem cell transplantation in multiple myeloma. Am. J. Hematol..

[34] Lee R., Storb R., Little M.T., Joslyn A., Spector M., Kuhr C.S. (2002). Percutaneous central dual-lumen catheter for apheresis in the canine. J. Investig. Surg. Off. J. Acad. Surg. Res..

[35] Kim S., Hosoya K., Kobayashi A., Okumura M. (2019). Comparison of three mobilization protocols for peripheral blood stem cell apheresis with Spectra Optia continuous mononuclear cell protocol in healthy dogs. Vet. Comp. Oncol..

[36] Abugomaa A., Elbadawy M. (2020). Patient-derived organoid analysis of drug resistance in precision medicine: Is there a value?. Expert Rev. Precis. Med. Drug Dev..

[37] Perdue J.J., Chandler L.K., Vesely S.K., Duvall D.S., Gilcher R.O., Smith J.W., George J.N. (2001). Unintentional platelet removal by plasmapheresis. J. Clin. Apher..

[38] Lee G., Arepally G.M. (2012). Anticoagulation techniques in apheresis: From heparin to citrate and beyond. J. Clin. Apher..

[39] Lydon H., Brooks R., McCaskie A., Henson F. (2018). Peripheral mononuclear blood cell apheresis in a preclinical ovine model. BMC Vet. Res..

